# Efficacy and safety of self-made Jianpi Huoxue Jieyu formula in treating ischemic stroke-related insomnia with Qi deficiency and blood stasis: A randomized controlled trial

**DOI:** 10.1097/MD.0000000000043514

**Published:** 2025-08-01

**Authors:** Jianhua Zhao, Libo Gao, Yudou Kang, Qisheng Zhang, Tianlin Sun, Jingxia Zhang

**Affiliations:** a Changjiaxiang Out-Patient Department, Affiliated Hospital of Gansu University of Chinese Medicine, Lanzhou City, Gansu Province, China; b Encephalopathy Department 1, Affiliated Hospital of Gansu University of Chinese Medicine, Lanzhou City, Gansu Province, China; c Cardiovascular and Cerebrovascular Diseases Department, Lintao Hospital of Traditional Chinese Medicine, Dingxi City, Gansu Province, China; d Child and Adolescent Psychology Department, Gansu Second People’s Hospital, Lanzhou City, Gansu Province, China.

**Keywords:** curative effect, insomnia, ischemic stroke, mechanism, Qi deficiency and blood stasis, self-made Jianpi Huoxue Jieyu formula

## Abstract

**Background:**

Insomnia commonly co-occurs with ischemic stroke (IS), especially in patients with Qi deficiency and blood stasis, but effective treatments are limited. This study examines the impact of a self-made Jianpi Huoxue Jieyu formula on IS-related insomnia, aiming to provide insights for its pharmacological management.

**Methods:**

Ninety IS patients with Qi deficiency and blood stasis accompanied by insomnia were randomly assigned to 2 groups (45 each) at the Affiliated Hospital of Gansu University of Chinese Medicine (2022–2023). The control group received standard treatment, while the observation group received additional treatment with the Jianpi Huoxue Jieyu formula. Clinical outcomes were evaluated using traditional Chinese medicine symptom scales, the Pittsburgh Sleep Quality Index (PSQI), and NIH Stroke Scale (NIHSS). Serum levels of hs-CRP, IL-6, TXB2, PAF, 5-HT, BDNF, and NGF were measured to explore the underlying mechanisms.

**Results:**

After treatment, traditional Chinese medicine symptom scores, PSQI, NIHSS scores, and levels of hs-CRP, IL-6, TXB2, and PAF were significantly lower in the observation group than in the control group, while levels of 5-HT, BDNF, and NGF were higher (*P* < .05). The clinical efficacy in the observation group was superior to that in the control group (*P* < .05).

**Conclusion:**

The Jianpi Huoxue Jieyu formula is safe and effective for treating IS-related insomnia due to Qi deficiency and blood stasis, potentially by reducing inflammation and enhancing neurological function and sleep quality.

## 1. Introduction

Ischemic stroke (IS) is a prevalent cerebrovascular disease with a close association with pathological processes such as atherosclerosis and thrombosis.^[1]^ It exhibits high morbidity, disability, mortality rates, and poor prognosis.^[2]^ The primary mechanism underlying this disease involves the occlusion of local cerebral arteries, leading to damage in the corresponding brain tissue area due to ischemia and hypoxia.^[3]^ Therefore, the core objective of the treatment strategy is to swiftly restore blood supply to the ischemic area, promoting the recovery and reconstruction of damaged nerve function.^[1]^ Within the Traditional Chinese Medicine (TCM) system, IS falls under the category of “stroke.”^[4]^ Its pathogenesis often involves an imbalance in qi and blood circulation, particularly the state of qi deficiency and blood stasis. This imbalance refers to inadequate or excessive consumption of qi and the abnormal transformation of blood, resulting in obstructed blood flow, brain dystrophy, and stroke.^[5,6]^

Insomnia pertains to the recurrent struggle for individuals to commence and sustain sleep for the majority of the nighttime hours over an extended period. It is characterized by troubles in initiating sleep and disturbances in sleep continuity, significantly influencing the individual’s daytime mental acuity and emotional stability.^[7]^ Numerous domestic and foreign studies have revealed that for the elderly population, insomnia is not only a single manifestation of the decline in sleep quality, but also an important factor that aggravates serious health risks such as cardiovascular and cerebrovascular diseases.^[8,9]^ Existing studies have clearly pointed out that insomnia is quite common in patients with IS and has become one of its main complications.^[10]^ Studies have shown that up to 57% of stroke patients have severe sleep problems, which further strengthens the inextricable link between sleep quality and IS.^[11]^

Insomnia following a stroke not only occurs as a part of stroke sequelae but also engages in complex interactions with stroke symptoms.^[12]^ This interplay mutually exacerbates the overall health status of patients. According to the TCM theory, the root cause of insomnia is associated with dysfunction in organs such as the heart, liver, and spleen, particularly an imbalance in yin and yang, a restless mind, and weakened spleen and stomach function, resulting in qi and blood deficiency.^[13]^ In stroke patients, insomnia is often induced by psychological stress stemming from poststroke sequelae, as well as by long-term qi and blood deficiencies and stagnation. Especially the spleen and stomach, as the key to the formation of qi and blood, its functional status directly affects the filling and circulation of qi and blood, which is an important pathological basis for qi deficiency and blood stasis and insomnia.^[14,15]^

The objective of this study was to explore the medical repercussions of a self-made Jianpi Huoxue Jieyu formula for treating IS with insomnia characterized by qi deficiency and blood stasis. The study aimed to construct a comparative group and utilize objective and quantitative assessment tools including the TCM symptom quantitative score, Pittsburgh Sleep Quality Index (PSQI), and National Institutes of Health Stroke Scale (NIHSS) to systematically monitor the improvement of symptoms, changes in sleep quality, and neurological function recovery before and after treatment. Furthermore, the study intended to monitor the alterations in blood biochemical indicators and nerve cell factors. By employing a range of scientific and rigorous research methods, this study aspires to furnish robust data support and a theoretical foundation in the treatment of IS with insomnia characterized by qi deficiency and blood stasis.

## 2. Object and Methods

### 2.1. Object

A comprehensive selection of 90 patients diagnosed with IS accompanied by insomnia of qi deficiency and blood stagnation type, admitted to Affiliated Hospital of Gansu University of Chinese Medicine, between 2022 and 2023, was conducted. These patients were subsequently randomized into 2 distinct groups: a control group comprising 45 cases and an observation group with an equal number of 45 cases. The Institutional Review Board of our hospital gave ethical approval to this research.

### 2.2. Criteria for diagnosis, inclusion, exclusion and removal

#### 2.2.1. Diagnostic criteria

Diagnostic criteria for insomnia: Presence of abnormal sleep and sleep-related daytime symptoms (fatigue, inattention, irritability), the above symptoms occur 3 times a week or more.

The TCM diagnostic criteria: according to the “diagnostic and therapeutic evaluation criteria for stroke (Trial)” syndrome differentiation for qi deficiency and blood stasis syndrome. The main symptoms were hemiplegia, confusion, poor speech or inability to speak, hemidysesthesia, and crooked mouth and crooked tongue. The secondary symptoms were headache, vertigo, drinking water choking, eye deviation, and ataxia.

Western diagnostic criteria: meet the diagnostic criteria for acute stroke, the diagnostic criteria for acute stroke include the following symptoms: Weakness or numbness on one side of the limb; one side of facial numbness or mouth skewed, speech is not clear; Fixed gaze to one side; Vision loss or blurriness in one or both eyes; Dizziness accompanied by vomiting; Previous rare episodes of severe headache and vomiting; Disturbance of consciousness or convulsions. If any symptoms suddenly appear, the possibility of stroke should be considered.

#### 2.2.2. Inclusion criteria^[16]^

Patients who met the criteria of “2023 Guidelines on the Diagnosis and Treatment of Insomnia in Adults-Brazilian Sleep Association ”^[17]^ and PSQI > 7; Conform to the diagnosis of acute stroke^[18]^; Conform to the diagnostic criteria of TCM syndrome differentiation; Those who had not used sedative hypnotic drugs in the past, or discontinued for more than 1 month.

#### 2.2.3. Exclusion criteria^[16]^

patients with insomnia caused by drugs, alcohol or secondary insomnia caused by organic diseases; patients who were taking or had taken drugs affecting the symptoms of the disease within 1 month; patients with a history of mental illness; patients with allergies to the drug components in this study; patients who are participating in other clinical drug trials.

#### 2.2.4. Criteria for elimination or shedding of cases

Patients with drug intolerance and large fluctuations in blood pressure and heart rate were excluded; Patients with poor treatment compliance or self-use of drugs that interfere with this study and affect the judgment of this test were excluded; Patients who did not use drugs according to the plan after inclusion or who were naturally separated, lost to follow-up, and automatically withdrew during the study were all shedding cases.

### 2.3. Intervention method

Control group: The subjects in this group were given basic treatment to prevent the recurrence of cerebrovascular diseases, such as regulating blood pressure, blood glucose and blood lipids, improving circulation, low-salt and low-fat diet, stabilizing plaques, and anti-platelet aggregation.

Observation group: Based on the treatment of the control group, an additional TCM treatment was introduced using a self-made Jianpi Huoxue Jieyu formula (Sichuan Xinhehua Chinese Medicine Decoction Pieces Co., Ltd.). One dose of the prescription was prepared daily by the pharmacy department of Affiliated Hospital of Gansu University of Chinese Medicine and the treatment involved boiling each dose with 500 mL of water until it was concentrated to 300 mL. The dose was then taken twice daily, in the morning and evening. Both groups of patients underwent continuous treatment for 4 weeks.The composition of the self-made Jianpi Huoxue Jieyu formula is shown in Table 1.

**Table 1:** Ingredients of Jianpi Huoxue Jieyu formula self designed.

Chinese name	English name	Latin botanical name	Dosage (g)
Huangqi	Milkvetch Root	Hedysarum Multijugum Maxim.	20
Danshen	Danshen Root	Salviae Miltiorrhizae Radix et Rhizoma	10
Taizishen	Heterophylly Falsestarwort Root	Pseudostellariae Radix	15
Duanlonggu	Drgon bones	Fossilia OssiaMastodi	30
Chaihu	Chinese Thorowax Root	Bupleuri Radix	10
Baizhu	Largehead Atractylodes Rh	Atractylodis Macrocephalae Rhizoma	15
Fuling	Indian buead	Poria	15
Duanmuli	Common Oyster Shell	Ostreae Concha	30
Yujin	Aromatic Turmeric Root-tuber	Curcumae Radix	12
Xiangfu	Nutgrass Galingale Rhizome	Cyperi Rhizoma	12
Yanhusuo	Corydalis Yanhusuo	Corydalis Rhizoma	10
Danggui	Chinese Angelica	Angelicae Sinensis Radix	20
Guizhi	Cmnamomi Mmulus	Cinnamomi Ramulus	10
Suanzaoren	Spine Date Seed	Ziziphus jujuba Mill. var. Spinosa	30
Wuweizi	Schisandrae Chinensis Fructus	Schisandra chinensis (Turcz.) Baill.	15
Gancao	liquorice root	Glycyrrhizae Radix et Rhizoma	10

### 2.4. Observation of indicators

#### 2.4.1. TCM symptom score

According to the diagnostic criteria of stroke qi deficiency and blood stasis syndrome, the TCM syndrome scores before and after treatment were determined. Main symptoms: hemiplegia; confusion; poor speech or inability to speak; hemidysesthesia; mouth and tongue deviation. Secondary symptoms: headache; vertigo; pupil changes; drinking water choking; eye deviation; ataxia; dim tongue, white greasy tongue coating or tooth marks; thick pulse. The scoring is based on the severity of the main symptoms, ranging from mild to severe: asymptomatic is scored as 0, mild symptoms are scored as 1, moderate symptoms are scored as 2, and severe symptoms are scored as 3. The lower the score, the lighter the symptoms.

#### 2.4.2. PSQI score

The PSQI scoring methodology was applied, adhering to the 7 constituent elements outlined in the PSQI scale.^[19]^ Each component is evaluated on a scale of 0 to 3 points, culminating in a total score that spans from 0 to 21 points. A higher cumulative score signifies a deterioration in sleep quality. The PSQI evaluation was administered under the supervision of a medical professional and typically required 5 to 10 minutes to be fully completed.

#### 2.4.3. NIHSS score

Based on NIHSS evaluation of neurological deficits before and after treatment,^[20]^ the total score on the scale was 42 points, with 21 to 42 points indicating severe neurological impairment, 16 to 21 points indicative of moderate to severe injury, 5 to 16 points indicative of moderate injury, 2 to <5 points indicative of mild or minor stroke, and <2 points indicating normal or almost normal neurological function. A higher score signifies more severe neurological impairment.

#### 2.4.4. Detection of serum-related factors

Prior to and subsequent to the treatment, a 5 mL sample of fasting blood was procured in the morning hours. This sample was promptly centrifugal to isolate the serum. Subsequent measurements were conducted on the serum to quantify the concentrations of hypersensitive C-reactive protein (hs-CRP), interleukin-6 (IL-6), thromboxane-B2 (TXB2), platelet-activating factor (PAF), serotonin (5-hydroxytryptamine, 5-HT), brain-derived neurotrophic factor (BDNF), and nerve growth factor (NGF).

#### 2.4.5. Adverse reactions

The incidence of adverse reactions during treatment was recorded in the 2 groups.

### 2.5. Statistical analysis method

Utilizing SPSS 28.0 software, a statistical analysis was conducted. For normally distributed measurement data, the values are presented as mean accompanied by standard deviation (x ± s). Comparisons of PSQI and NIHSS scores, both before treatment and 4 weeks posttreatment, as well as changes in levels of hs-CRP, IL-6, TXB2, PAF, 5-HT, BDNF, and NGF over the same timeframe, between the study and control groups, were accomplished using the *t*-test. The Mann–Whitney *U* test was employed to assess the disparity in the potency of TCM symptoms between the 2 groups. A statistical significance threshold was set at a *P*-value of < .05.

## 3. Result

### 3.1. General data analysis

The present study initiated with a total enrollment of 90 patients. However, a subsequent attrition occurred, with 4 patients from the control group exiting the study; 3 of these withdrawals were due to a deterioration in their condition necessitating a shift in treatment strategy, while 1 patient voluntarily withdrew. In the observation group, 3 cases were lost to follow-up, 2 because they became untraceable, and 1 due to adverse reactions leading to treatment discontinuation. The comprehensive demographic and clinical characteristics of the study participants are summarized in Table 2. No noteworthy variations were seen between the 2 groups in terms of gender, age, BMI, educational attainment, or illness length (*P* > .05).

**Table 2:** Participate in the general data statistics of patients.

Characteristics		Control	Observation	*t*/*Z*/χ^*2*^	*P*
Gender	Male	19	20	0.082	.774
	Female	22	22		
Age		57.36 ± 7.41	57.34 ± 6.36	0.015	.988
BMI (kg/m^2^)		23.05 ± 2.89	23.91 ± 2.98	1.338	.185
Educational level	Primary school	16	11	−0.952	.341
	Junior high school	10	14		
	Senior high school	10	10		
	Junior college and above	5	7		
Course of disease (d)		96.88 ± 44.75	108.60 ± 44.28	0.244	1.199

### 3.2. Comparison of TCM symptom scores

After undergoing treatment, both groups exhibited reduced TCM symptom scores pertaining to hemiplegia, confusion, impaired speech or aphasia, and mouth and tongue deviation, as compared to their pretreatment scores. Notably, the observation group demonstrated a significantly more pronounced decrease in TCM symptom scores compared to the control group (*P* < .05), as evident from the data presented in Table 3 (hemiplegia; confusion; poor speech or inability to speak; mouth and tongue deviation).

**Table 3:** Comparison of traditional Chinese medicine syndrome scores in both groups.

Group	N	Pretreatment/post-treatment	1	2	3	4
Control	41	Pretreatment	2.50 ± 0.32	2.40 ± 0.31	2.39 ± 0.48	2.45 ± 0.29
		Post-treatment	1.57 ± 0.26*	1.52 ± 0.35*	1.41 ± 0.27*	1.52 ± 0.42*
Observation	42	Pretreatment	2.56 ± 0.31	2.28 ± 0.46	2.58 ± 0.21	2.36 ± 0.53
		Post-treatment	1.06 ± 0.24*†	0.90 ± 0.43*†	0.99 ± 0.08*†	0.98 ± 0.17*†

* Compared to the same group pretreatment, *P* < .05.

† Compared to the posttreatment control group, *P* < .05.

### 3.3. Comparison of total sleep quality and neurological deficit score

Following treatment, statistical analysis was performed on the data from both patient groups using *t*-tests. The findings revealed that both the total PSQI scores and NIHSS scores had significantly decreased in both groups compared to their pretreatment levels (*P* < .05). Furthermore, the observation group exhibited notably lower total PSQI scores and NIHSS scores than the control group (*P* < .01). These outcomes are summarized in Table 4.

**Table 4:** PSQI total score and NIHSS comparison table between the 2 groups of patients.

Group	N	Pretreatment/post-treatment	PSQI	NIHSS
Control	41	Pretreatment	13.68 ± 0.95	19.22 ± 4.12
		Post-treatment	9.00 ± 0.57*	12.76 ± 3.86*
Observation	42	Pretreatment	13.47 ± 0.87	18.92 ± 4.12
		Post-treatment	6.06 ± 0.64*†	7.56 ± 2.69*†

NHSS = National Institutes of Health Stroke Scale, PSQI = Pittsburgh Sleep Quality Index.

* Compared to the same group pretreatment, *P* < .05.

† Compared to the posttreatment control group, *P* < .05.

### 3.4. The levels of hs-CRP, IL-6, TXB2 and PAF in serum were compared

Following treatment, the serum concentrations of hs-CRP, IL-6, TXB2, and PAF were observed to have diminished in both patient groups in comparison to their pretreatment values. Notably, the observation group exhibited a statistically significant lowering of serum levels for hs-CRP, IL-6, TXB2, and PAF in comparison to the control group, with the difference being statistically significant (*P* < .05). These findings are displayed in Table 5.

**Table 5:** A comparison of serum-related factors’ expression levels between the 2 groups is presented.

Group	N	Pretreatment/post-treatment	hs-CRP (mg/L)	IL-6 (ng/L)	TXB2 (ng/L)	PAF (ng/L)
Control	41	Pretreatment	29.88 ± 2.86	8.88 ± 2.53	74.71 ± 2.35	95.34 ± 6.48
		Post-treatment	9.73 ± 3.10*	5.04 ± 2.04*	55.04 ± 2.14*	74.79 ± 4.96*
Observation	42	Pretreatment	29.74 ± 2.29	8.91 ± 2.31	75.48 ± 2.96	96.03 ± 2.70
		Post-treatment	5.77 ± 1.41*†	3.56 ± 1.74*†	40.28 ± 2.49*†	61.25 ± 4.33*†

hs-CRP = hypersensitive C-reactive protein, IL-6 = interleukin 6, PAF = platelet-activating factor, TXB2 = thromboxane B2.

* Compared to the same group pretreatment, *P* < .05.

† Compared to the posttreatment control group, *P* < .05.

### 3.5. Comparison of serum nerve cell factor expression levels

Posttreatment, there was a marked elevation in serum concentrations of 5-HT, BDNF, and NGF in both groups when compared to pretreatment (*P* < .05). Furthermore, the observation group exhibited significantly higher levels of these neurotransmitters and growth factors in their serum compared to the control group, with the difference being statistically significant (*P* < .05) referring to Table 6.

**Table 6:** Comparison of BDNF and NGF between the 2 groups.

Group	N	Pretreatment/post-treatment	5-HT (μg/L)	BDNF (μg/L)	NGF (ng/L)
Control	41	Pretreatment	74.22 ± 4.25	8.11 ± 1.41	9.17 ± 2.35
		Post-treatment	89.99 ± 3.88*	11.47 ± 2.58*	11.83 ± 1.32*
Observation	42	Pretreatment	76.48 ± 2.98	7.95 ± 1.76	9.16 ± 1.93
		Post-treatment	109.27 ± 4.08*†	15.56 ± 2.65*†	15.22 ± 3.17*†

BDNF = brain-derived neurotrophic factor, 5-HT = 5-hydroxytryptamine, NGF = nerve growth factor.

* Compared to the same group pretreatment, *P* < .05.

† Compared to the posttreatment control group, *P* < .05.

### 3.6. Safety index analysis

Before and after treatment, the vital signs of the 2 groups were stable, and there were no obvious adverse reactions. In the observation group, 1 patient had dyspepsia, but the symptoms were mild and did not require specific treatment. In addition, the safety indicators such as blood test (three routine, biochemical, etc), electrocardiogram and other tests showed no obvious abnormalities, and there were no significant side effects and other abnormalities during the clinical study.

## 4. Discussion

Scientific studies have clearly pointed out that long-standing sleep disorders can significantly affect the body’s hormone levels and limit the normal synthesis of intracellular chemicals. In extreme cases, such disorders may aggravate or directly promote a series of cardiovascular and cerebrovascular diseases, including coronary heart disease, hypertension, myocardial infarction, and stroke.^[21,22]^ And long-term insomnia may damage the immune system and nerve function, increase pain sensitivity, lead to depression, and other negative emotional states, thereby delaying recovery and severely impacting patients’ quality of life.^[23]^ However, the current clinical treatment methods mostly rely on traditional sedative and sleeping drugs, aiming to slow down the process of stroke by relieving insomnia symptoms. However, the long-term effect of this method is limited, and patients may face side effects such as drug dependence, memory loss, and palpitation.^[24–26]^ Consequently, there is a critical need to explore safer and more effective treatment strategies.

IS is one of the frequently occurring cerebrovascular diseases among the middle-aged and elderly population, characterized by a high risk of complications and disability rates.^[27]^ When this condition occurs, blood flow to the brain is obstructed, causing damage to brain cells due to lack of oxygen, leading to a series of neurological dysfunctions, such as loss of motor ability (paralysis), speech communication disorders, and sensory abnormalities.^[18,28]^ In the TCM clinical differentiation, the syndrome of qi deficiency and blood stasis poststroke is a common type, with core symptoms closely related to these 2 pathological states.^[29]^ According to TCM theory, the root of IS with insomnia symptoms in the qi deficiency and blood stasis type is deeply rooted in the brain, often affecting patients who are middle-aged or older, with deficiencies in both qi and blood. Qi, as the driving force of life activities, facilitates smooth blood circulation when sufficient; however, qi deficiency weakens this driving force, resulting in blood stagnation in the brain’s vessels, ultimately triggering the condition.^[16,30]^

The TCM further points out that the occurrence of stroke is mostly derived from emotional disorders, external evils, imbalance of work and rest, excessive diet and other factors. These factors collectively disrupt the body’s organ functions, leading to poor qi and blood circulation, qi deficiency, and blood stasis, which ultimately obstruct the brain’s collaterals and cloud the spirit.^[1,31]^ Therefore, the fundamental strategy for treating such conditions is to tonify qi, invigorate blood, and resolve stasis, aiming to restore normal qi and blood flow and clear the brain’s collaterals. In the course of treatment, the TCM emphasizes reconciling viscera qi and blood, restoring the body’s yin and yang balance, which is the key to achieve good therapeutic effect. As stated in the ancient text “Clinical Guidelines for Medical Cases,” quality sleep is a natural manifestation of the body’s yang qi returning to its proper place and the spirit finding peace.^[32,33]^ Additionally, given that spleen deficiency can affect nutrient absorption and distribution, disrupting yin-yang balance, special attention should be paid to tonifying spleen qi during treatment to promote the recovery and harmonious coexistence of the heart and spleen, thereby improving the patient’s overall health.^[13]^

The therapeutic approach in this study involves a combination of multiple compounds. Understanding the nature of these interactions is crucial for elucidating the mechanism of action and ensuring the safety and efficacy of the treatment. The Jianpi Huoxue Jieyu formula is based on TCM principles, emphasizing the synergistic effects of multiple herbs working together to achieve therapeutic outcomes. This formula comprises a variety of herbs, each with its own pharmacological properties, collectively aiming to tonify the spleen, invigorate blood, resolve stasis, and calm the mind. The core ingredients, Taizishen and Huangqi, serve as the monarch herbs. Taizishen focuses on replenishing heart qi, while Huangqi emphasizes tonifying the spleen and boosting qi; together, they nourish both heart yin and yang fundamentally, enhancing cardiac function. Danshen and Guizi act as ministerial herbs, with Danshen providing effects that invigorate blood, dispel stasis, and calm the spirit, while Guizi warms yang and unblocks the meridians, facilitating qi circulation and further promoting blood flow. The supporting herbs in the formula are diverse: Baishu and Fuling work together to promote diuresis and dampness drainage, Caihu aids in soothing the liver and resolving depression, and Duanmuli and Duanlonggu have the functions of calming the spirit and preserving yang. Additionally, Yujin promotes blood circulation and resolves depression, while Xiangfu and Yanhusuo together facilitate liver soothing, depression relief, and pain alleviation. Danggui addresses both blood activation and supplementation, enhancing the overall therapeutic effect. Furthermore, Suanzaoren, Wuweizi, and Gancao are added. Suanzaoren, first recorded in “Jinkui Yaolue,” treats deficiency and worry leading to insomnia. Wuweizi influences both inhibition and excitation in the cerebral cortex, achieving balance. Gancao is used to harmonize all the formula. The whole prescription is used together to give full play to the functions of tonifying spleen and invigorating qi, promoting blood circulation and removing blood stasis, warming meridians and dredging meridians, soothing liver and relieving depression, calming heart and tranquilizing mind.The entire prescription is designed to fully utilize its functions: tonifying the spleen, replenishing qi, invigorating blood, resolving stasis, warming and unblocking the meridians, soothing the liver and alleviating depression, as well as calming the heart and calming the mind.

The findings of this research underscore that, in contrast to conventional western medical interventions, the application of the customized remedy aimed at strengthening the spleen, enhancing blood circulation, and alleviating depressive symptoms in the observation group yielded notable efficacy advantages. Notably, there was a marked decrease in the TCM symptom scores, indicating the efficacy of the combined treatment in managing IS patients with qi deficiency and blood stasis, thereby optimizing clinical outcomes and mitigating TCM symptoms. Moreover, subsequent to a 4-week treatment period, the observation group exhibited lower NIHSS and PSQI total scores compared to the control group, emphasizing the capacity of this formula to substantially mitigate neurological deficits and promote better sleep quality among these patients. Although there are some preliminary observations and traditional knowledge supporting the synergistic effects of the herbs in the Jianpi Huoxue Jieyu formula, comprehensive systematic studies are still lacking. The interactions between multiple compounds can be highly complex, potentially involving both additive and synergistic effects, which necessitate sophisticated experimental designs and analytical methods to fully elucidate. It is worth noting that understanding the absorption, distribution, metabolism, and excretion characteristics as well as the dose–response relationships of the drugs in the Jianpi Huoxue Jieyu formula is crucial for optimizing its therapeutic efficacy and safety. Currently, pharmacokinetic and pharmacodynamic studies on individual herbs and their combinations within this formula are still relatively limited. When used in combination, the various components in the formula may alter each other’s pharmacokinetic profiles through mechanisms such as affecting absorption or competing for metabolic enzymes or transporters, resulting in synergistic or antagonistic pharmacological effects. It is recommended that future research employ techniques such as liquid chromatography-mass spectrometry to systematically investigate the in vivo processes of the active components, establish dose-efficacy-toxicity relationship curves, and clarify the effective dose ranges and safety thresholds. This will provide a scientific basis for optimizing the formula.

The primary and most prevalent cause of IS is atherosclerosis, which involves an inflammatory response throughout its progression and serves as the foundation for thrombotic diseases.^[34]^ hs-CRP and IL-6 serve as nonspecific markers of inflammation, effectively indicating the diagnosis and prevention of cardiovascular and cerebrovascular events. They are crucial in diagnosing and preventing conditions such as coronary heart disease, stroke, and peripheral vascular embolism^[35]^; TXB2 contributes to platelet aggregation and vasoconstriction,^[36]^ while PAF, a class of intracellular phospholipid metabolites, activates platelets and mediates inflammation.^[37]^ Additionally, 5-HT, an essential neurotransmitter in the cerebral cortex and synapses, which inhibits the release of dopamine and can induce feelings of pleasure. Studies show that abnormal serum 5-HT levels in patients with IS and insomnia are significantly related to decreased sleep quality.^[38]^ Serum NGF is an important factor to promote nerve cell growth and nerve repair, and its level expression can directly reflect the recovery of nerve function.^[39]^ BDNF stands as a pivotal member within the neurotrophic factor family, predominantly residing in the hippocampus and cortex regions. It assumes a vital function in fostering neuronal survival within the intricate landscape of the central nervous system.^[40]^

The outcomes of this investigation reveal that while serum hs-CRP and IL-6 concentrations declined in the control group posttreatment, the application of the self-made Jianpi Huoxue Jieyu formula led to even more pronounced reductions in the observation group, achieving statistically significant differences. This finding implies that the formula promotes the breakdown of inflammatory biomarkers and mitigates inflammatory harm. Furthermore, the observation group displayed notably lower levels of PAF and TXB2 posttreatment compared to the control group, suggesting that the formula amplifies therapeutic benefits by lessening the severity of thrombosis. Both cohorts experienced elevated plasma 5-HT levels post-intervention, yet the observation group exhibited a marked divergence from the control group, indicative of the formula’s ability to modulate central neurotransmitters and bolster brain injury recovery, thereby enhancing sleep quality. Additionally, BDNF and NGF concentrations were significantly augmented in the observation group, emphasizing the therapeutic potency of the formula for IS patients experiencing insomnia due to qi deficiency and blood stasis. The observed results imply that this therapeutic approach holds efficacy in addressing IS and insomnia characterized by qi deficiency and blood stasis, favorably influencing neurotrophic factors, accelerating the rehabilitation of neurological and motor capabilities, subsequently enhancing patients’ ability to perform daily activities.

Prior to clinical use, there are no clear and comprehensive reports on preclinical safety and toxicity studies of the self-formulated Jianpi Huoxue Jieyu Formula, particularly regarding acute, subchronic, or chronic toxicity studies in animal models. However, preclinical studies on similar formulations have provided some relevant data. One study demonstrated that a similar Jianpi Huoxue granules formulation exhibited liver-protective effects in animal models. In a rat model of nonalcoholic fatty liver disease induced by a methionine-choline-deficient diet, Jian-Pi Huo-Xue significantly reduced serum alanine aminotransferase and aspartate aminotransferase levels, decreased the accumulation of triglycerides and total cholesterol in the liver, and improved liver inflammation.^[41]^ Another study investigated the therapeutic effects of a similar Jianpi Jiedu Recipe on colon cancer both in vitro and in vivo. The study found that this formula displayed good inhibitory effects on various colon cancer cells in vitro and showed favorable therapeutic outcomes in a nude mouse xenograft tumor model. Meanwhile, it did not significantly affect the liver index and spleen index of the nude mice, indicating low toxicity.^[42]^ In clinical practice, no obvious adverse reactions were observed with the self-formulated Jianpi Huoxue Jieyu Formula in this study. Only one patient experienced mild dyspepsia, which did not require special treatment. This suggests that the formula has good tolerability in clinical applications. Before advancing to human trials or applying it as a therapeutic agent for any disease, it is of utmost importance to confirm that these preclinical safety evaluations are in alignment with the established regulatory guidelines for drug development, such as those of the FDA and EMA. To further ensure its safety in humans, this study will undertake more comprehensive preclinical toxicity studies, encompassing acute, subchronic, and even chronic toxicity trials.

## 5. Concussion

In conclusion, the combined use of the self-made Jianpi Huoxue Jieyu formula has demonstrated significant effectiveness in treating IS of qi deficiency and blood stasis type accompanied with insomnia. This approach notably enhances neurological function, improves sleep quality, manages the disease progression, and minimizes the risk of adverse effects, all while maintaining a high level of safety. These findings offer strong evidence for the application of TCM in addressing IS accompanied by insomnia due to qi deficiency and blood stasis. However, evaluating the long-term safety and sustained efficacy of the Jianpi Huoxue Jieyu Formula is of great necessity, especially for chronic diseases like IS-related insomnia that require long-term treatment. Current studies mainly offer insights into short-term effects, lacking long-term data spanning several months to years. Since side effects may only emerge after prolonged use, and efficacy may diminish due to factors such as drug tolerance, we are committed to conducting comprehensive long-term studies and post-marketing surveillance to ensure its continued safety and effectiveness, thereby providing robust evidence for its clinical use.

## Author contributions

**Conceptualization:** Yudou Kang, Qisheng Zhang.

**Data curation:** Libo Gao, Tianlin Sun.

**Writing – original draft:** Jianhua Zhao, Jingxia Zhang.

**Writing – review & editing:** Jianhua Zhao.
